# Association of zinc serum level with metabolic syndrome in iranian children and adolescents: The CASPIAN-V study

**DOI:** 10.3389/fnut.2022.932746

**Published:** 2022-08-09

**Authors:** Mostafa Qorbani, Negar Movasaghi, Nami Mohammadian Khonsari, Elnaz Daneshzad, Gita Shafiee, Haleh Ashraf, Leily Sokoty, Armita Mahdavi-Gorabi, Mehdi Ebrahimi, Ramin Heshmat, Roya Kelishadi

**Affiliations:** ^1^Non-communicable Diseases Research Center, Alborz University of Medical Sciences, Karaj, Iran; ^2^Chronic Diseases Research Center, Endocrinology and Metabolism Population Sciences Institute Tehran University of Medical Sciences, Tehran, Iran; ^3^Dietary Supplements and Probiotic Research Center, Alborz University of Medical Sciences, Karaj, Iran; ^4^Research Development Center, Sina Hospital, Tehran University of Medical Sciences, Tehran, Iran; ^5^Cardiac Primary Prevention Research Center, Cardiovascular Diseases Research Institute, Tehran University of Medical Sciences, Tehran, Iran; ^6^Social Determinants of Health Research Center, Alborz University of Medical Sciences, Karaj, Iran; ^7^Department of Internal Medicine, Faculty of Medicine, Sina Hospital, Tehran University of Medical Sciences, Tehran, Iran; ^8^Child Growth and Development Research Center, Research Institute for Primordial Prevention of Non-communicable Disease Isfahan University of Medical Sciences, Isfahan, Iran

**Keywords:** metabolic syndrome, zinc, adolescents, children, CASPIAN-V

## Abstract

**Introduction:**

Metabolic syndrome comprises a set of metabolic risk factors associated with cardiovascular disease and type 2 diabetes. Zinc plays an essential role in numerous enzyme functions that may be associated with metabolic dysfunctions. The relationship between serum zinc levels and metabolic syndrome in adolescents has not been specifically studied. Therefore, this study was performed to determine the relationship between serum zinc levels and metabolic syndrome in Iranian children and adolescents.

**Materials and methods:**

This cross-sectional study was performed using data collected in the CASPIAN-V study. In this project, data were collected using interviews, examinations, biochemical assessments, anthropometric studies, and the nutritional status of participants. The variables considered in this study included serum zinc levels, triglycerides (TG), low-density lipoprotein (LDL), high-density lipoprotein (HDL), fasting blood sugar, height, weight, abdominal circumference, and systolic and diastolic blood pressure.

**Results:**

A total of 1371 participants were included in this study, with a mean age of 12.24 ± 3.23 years. In total, 12.40% (*n* = 170) of the study population had metabolic syndrome, of which 55.7% were boys and 44.3% were girls. Mean zinc levels (μg/dL) in patients with and without metabolic syndrome were 107.03 and 110.6, respectively (*p*-value = 0.211) and 111.8 for boys and 109.10 for girls (*p*-value = 0.677).

**Conclusion:**

This cross-sectional study showed no association between serum zinc levels and metabolic syndrome in children. Further similar studies and cohort studies with large sample sizes are needed to reveal the exact relationship between serum zinc levels and metabolic syndrome.

## Introduction

Metabolic syndrome is a set of metabolic risk factors associated with cardiovascular diseases and type 2 diabetes (1). Numerous definitions of components and their diagnostic boundaries have been proposed for this syndrome, all of which are associated with metabolic disorders such as elevated blood pressure, impaired glucose and insulin metabolism, dyslipidemia, and central obesity (Measured parameters include: abdominal obesity or waist circumference equal to or more than 90th percentile of the age and sex; Systolic or diastolic blood pressure (SBP or DBP) equal to or greater than 90th percentile by height, age, and sex; Serum triglyceride (TG) level greater than 110 mg/dL, High-density lipoprotein (HDL) equal to or less than 40 mg/dL, fasting glucose level equal to or more than 100 mg/dL) (1–3). Metabolic syndrome is one of the main health hazards worldwide, resulting in a significant number of years lost (DALY) due to its associated morbidity and mortality (3, 4). Although many studies have been conducted on the prevalence of this syndrome in adults based on different definitions and its relationship with cardiovascular diseases (2, 3), there is no precise definition for it in childhood and adolescence. As nutrition plays a major role in growth and development in children, and deficiencies in various nutrients could result in metabolic defects (5), it seems that deficiencies in trace elements such as zinc which plays an essential role in numerous enzyme functions could result in metabolic dysfunction as well (6).

Zinc is a rare and essential element in the body involved in the metabolism of nucleic acids and their stability in protein synthesis, cell division, and gene expression. Furthermore, zinc plays an essential role in the activity of more than 300 enzymes in the body. Its deficiency can result in many skin diseases, mental disorders, pregnancy, lactation, growth disturbances, and susceptibility to infections (7). Hence, in theory, zinc deficiency can result in metabolism defects and metabolic disorders. Since the body does not have a proper zinc reserve, nutrition plays a significant role in providing this micronutrient (8).

Although severe zinc deficiency is rare, studies have shown that mild to moderate zinc deficiency occurs on a large scale in unbalanced diets (9). In Iran, due to the calcareous nature of agricultural soils, bicarbonate water, and the lack of zinc-containing fertilizers, the amount of zinc absorbed by plants is minimal (10–12).

The relationship between serum zinc levels and the incidence of metabolic syndrome in adolescents has not been specifically studied, and there has been controversy regarding the effects of zinc on metabolic syndrome (13). Therefore, this study was performed to determine the relationship between serum zinc levels and metabolic syndrome in children and adolescents.

## Materials and methods

### Study design

This cross-sectional study was performed using data collected in the Caspian-V study. (Caspian-V study, was a care system for health-related behaviors and risk factors for diseases in students in Iran).

### Data collection

Sampling was done by multi-stage using cluster and stratified sampling method. Class sampling was performed in each province of the country according to the student’s residence (city or village) and educational level (primary and secondary) in a manner commensurate with the size with an equal sex ratio. This means that the number of boys and girls in each province was equal, and their ratio in urban and rural areas was also proportional to the number of urban and rural students. Similarly, the number of samples was divided between the educational levels in the city and the village in proportion to the number of students studying at each level. In this study, 480 students were selected from each province (i.e., 48 clusters of 10 people in each province). In total, according to the study in 31 provinces, 14,880 people were surveyed using the standard questionnaire “WHO-GSHS,” which has been translated into Persian, and its validity has been evaluated and approved in previous studies (14). Trained professionals collected information about health-related behaviors and risk factors for diseases; and assessed anthropometric measurements, including height, weight, waist circumference, hip, neck, wrist circumferences, and blood pressure, for all participating students. One-third of the number of clusters from each province were randomly selected for blood sampling, and a skilled blood sampler took six ccs of their venous blood with consideration of all health issues to measure blood glucose indices, lipid profile, liver enzymes, and serum zinc level. It should be noted that the waist circumference was measured as a waist from the tangent and above the iliac crest to the ground with a metal meter. Metabolic syndrome was defined based on NHANES III (Third National Health And Nutrition Examination Survey)(15) as the presence of 3 out of 5 criteria for the diagnosis of this syndrome, including abdominal obesity or waist equal to or more than 90th percentile in terms of age and sex; SBP or DBP equal to or greater than 90th percentile by height, age, and sex; serum TG level greater than 110 mg/dL, HDL serum equal to or less than 40 mg/dL, fasting serum glucose levels equal to or greater than 100 mg/dL.

### Inclusion and exclusion criteria

The study population was children and adolescents studying in primary or secondary school whose information was recorded in the Caspian Cohort. Participants with incomplete data, according to the studied variables, as well as individuals with reduced renal function (glomerular filtration rate; GFR < 30), Chronic liver diseases, and glucocorticoids were excluded from the study.

## Data analysis

The SPSS software version 21 was used for data analyzes. Descriptive analysis results on quantitative variables are presented as mean and standard deviation, and qualitative variables as frequency and relative frequency. Furthermore, the correlation of coefficients was calculated. A *p*-value less than 0.05 was considered statistically significant.

## Ethical considerations

No information about the identities of individuals entered the study process; hence, the data were anonymous, and at no stage of the study, the individuals’ information was recorded or mentioned. Furthermore, this study was approved by the ethics committee of the Alborz University of Medical Sciences.

## Results

A total of 1,371 participants were included in this study, with a mean age of 12.24 ± 3.22 years. In total, 6.41% (*n* = 88) of the study population had metabolic syndrome, of which 55.68% were boys, and 44.32% were girls. The means of height, weight, waist circumference, SBP, DBP, TG and HDL were 147.24 ± 18.13 cm, 42.64 ± 16.5 kg, 67.7 ± 11.72 cm, 101.33 ± 13.46 mmHg, 65.45 ± 10.76 mmHg, 88.08 ± 44.58 mg/dL, and 46.88 ± 9.24 mg/dL, respectively. Serum zinc level mean was 107.23 ± 25.81 μg/dL. The presence or absence of metabolic syndrome based on demographic parameters has been categorized in Table 1. As shown in this table, no significant difference was observed between zinc levels in children and adolescents with or without metabolic syndrome (*p*-value = 0.211). Among the different variables among children with metabolic syndrome and others, only a significant difference in their TG levels can be seen. Participants with metabolic syndrome had greater TG and FBS compared to those without metabolic syndrome (109.29 ± 68.25 vs. 86.62 ± 41.18; *p*-value < 0.0001 and 99.26 ± 8.65 vs. 92.95 ± 8.32; *p*-value < 0.0001, respectively).

**TABLE 1:** Demographic status of the study population according to the presence or absence of metabolic syndrome (*n* = 1371).

Variables	Total	Metabolic syndrome		*P*-value
		Yes *N* = 88	No *N* = 1283	
**Gender;** n (%)				
Female	675 (49.2)	39 (5.8)	636 (94.2)	0.340
Male	696 (50.8)	49 (7.0)	647 (93.0)	
**Residential region** n (%)				
Rural	370 (27)	19 (5.1)	351 (94.9)	0.238
Urban	1001 (73.0)	69 (6.9)	932 (93.1)	
Age (year)	12.24 (3.22)	11.81 (3.23)	12.29 (3.24)	0.184
Weight (kg)	42.64 (16.50)	42.69 (15.90)	42.54 (16.41)	0.934
BMI (kg/m^2^)	18.99 (4.82)	19.23 (4.21)	18.96 (4.89)	0.618
WC (cm)	67.70 (11.72)	69.13 (12.79)	67.54 (11.76)	0.225
NC (cm)	30.23 (3.71)	30.01 (3.51)	30.26 (3.73)	0.531
Wrist Circumference (cm)	15.12 (2.11)	15.39 (2.36)	15.12 (2.11)	0.247
Hip Circumference (cm)	78.89 (14.99)	79.44 (15.77)	78.91 (14.96)	0.748
WHtR	0.46 (0.06)	0.47 (0.06)	0.46 (0.06)	0.136
SBP (mmHg)	101.33 (13.46)	101.45 (16.29)	101.35 (13.36)	0.947
DBP (mmHg)	65.45 (10.76)	64.81 (11.85)	65.46 (10.78)	0.589
TG (mg/dl)	88.08 (44.58)	109.29 (68.25)	86.62 (41.18)	**<0.0001**
Cholesterol (mg/dl)	152.95 (26.57)	154.27 (24.88)	152.51 (26.70)	0.550
LDL (mg/dl)	88.77 (22.60)	88.21 (21.10)	88.59 (22.62)	0.880
HDL (mg/dl)	46.88 (9.24)	45.78 (11.27)	46.88 (9.08)	0.283
FBS (mg/dl)	93.16 (8.64)	99.26 (8.65)	92.95 (8.32)	**<0.0001**
Serum Zinc (μ g/dL)	107.23 (25.81)	110.60 (29.84)	107.02 (25.59)	0.211
**ST n (%)**				
Low	1044 (76.1)	70 (6.7)	974 (93.3)	0.440
High	327 (23.9)	18 (5.5)	309 (94.5)	
**PA level n (%)**				
Low	471 (34.4)	33 (7.0)	438 (93.0)	0.327
Moderate	490 (35.7)	25 (5.1)	465 (94.9)	
High	410 (29.9)	30 (7.3)	380 (92.7)	
**SES n (%)**				
Low	370 (27.0)	23 (6.2)	347 (93.8)	0.732
Moderate	569 (41.5)	34 (6.0)	535 (94.0)	
High	432 (31.5)	31 (7.2)	401 (92.8)	

BMI, Body Mass Index; WC, Waist Circumference; WHtR, waist-to-height ratio; NC, Neck Circumference; SBP, systolic blood pressure; DBP, diastolic blood pressure; TG, Triglyceride; LDL, Low-density Lipoprotein; HDL, High-density Lipoprotein; FBS, Fasting Blood Sugar; ST, screen time (above 2 h per day are regarded as high, otherwise as low); PA, physical activity; SES, socioeconomic status.

Data presented as mean (SD) and number (%) for quantitative and qualitative variables, respectively.

SES,PA and other abbreviations are listed under the table or their definition can be found in the methods, or under the bolded variable.

Furthermore, there was no significant difference between zinc levels and sex in participants with and without metabolic syndrome. (*p*-value = 0.34). Similarly, there was no significant relationship between serum zinc levels and the age of children in children without metabolic syndrome (*p*-value = 0.184).

In Table 2, the association between metabolic syndrome components and dyslipidemia with serum zinc levels based on linear logistic regression analysis can be seen. No significant association was found between serum zinc levels and any metabolic syndrome components. Table 3 illustrates the same data but is based on serum zinc tertiles. As can be seen in Tables 2, 3, no significant relationship between serum zinc levels and the aforementioned variables was observed.

**TABLE 2:** Association between metabolic syndrome components and dyslipidemia with serum zinc level in children: linear regression analysis.

Variables	β	Standard error	*P*-value
**FBS (mg/dl)**			
Crude model	0.30	0.29	0.291
Adjusted model	0.23	0.29	0.419
Adjusted + BMI	0.23	0.29	0.417
**SBP (mmHg)**			
Crude model	0.08	0.47	0.853
Adjusted model	0.14	0.46	0.751
Adjusted + BMI	0.14	0.46	0.748
**DBP (mmHg)**			
Crude model	0.07	0.37	0.850
Adjusted model	0.17	0.38	0.649
Adjusted + BMI	0.17	0.37	0.640
**HDL (mg/dl)**			
Crude model	–0.13	0.31	0.666
Adjusted model	–0.07	0.31	0.804
Adjusted + BMI	–0.07	0.31	0.804
**LDL (mg/dl)**			
Crude model	–0.27	0.75	0.716
Adjusted model	–0.15	0.76	0.836
Adjusted + BMI	–0.15	0.76	0.839
**TG (mg/dl)**			
Crude model	1.09	1.49	0.463
Adjusted model	0.87	1.50	0.561
Adjusted + BMI	0.87	1.50	0.562
**TC (mg/dl)**			
Crude model	–0.10	0.89	0.907
Adjusted model	0.07	0.90	0.937
Adjusted + BMI	0.07	0.90	0.936
**WC (cm)**			
Crude model	0.16	0.41	0.692
Adjusted model	0.004	0.37	0.991

FBS, fasting blood sugar; SBP, systolic blood pressure; DBP, diastolic blood pressure; TG, triglyceride; TC, total cholesterol; LDL, low-density lipoprotein; HDL, high-density lipoprotein; WC, waist circumference.

**Adjusted model:** Variables were controlled for age, gender, socioeconomic status, physical activity, residential region, and screen time. Moreover, MetS components except for WC were adjusted for one more confounder (BMI).

**TABLE 3:** Association between metabolic syndrome components and dyslipidemia with tertiles of serum zinc in children: logistic regression analysis.

Variables	Serum Zinc tertiles			*P* trend
	1 (< 94)	2 (94–113)	3 (113 <)	
**MetS (*n* = 88)**				
Crude model	1	0.75 (0.43; 1.30)	1.08 (0.64; 1.80)	0.751
Adjusted model	1	0.73 (0.42; 1.27)	1.01 (0.60; 1.70)	0.938
**FBS ≥ 100 mg/dl (*n* = 54)**				
Crude model	1	1.87 (0.89; 3.91)	1.91 (0.91; 4.02)	0.097
Adjusted model	1	1.78 (0.84; 3.74)	1.61 (0.76; 3.43)	0.253
Adjusted + BMI	1	1.80 (0.85; 3.78)	1.62 (0.76; 3.45)	0.249
**SBP ≥ 90th (*n* = 77)**				
Crude model	1	1.15 (0.66; 1.98)	0.81 (0.45; 1.48)	0.526
Adjusted model	1	1.05 (0.60; 1.84)	0.76 (0.41; 1.41)	0.400
Adjusted + BMI	1	1.08 (0.61; 1.90)	0.77 (0.41; 1.43)	0.417
**DBP ≥ 90th (*n* = 209)**				
Crude model	1	1.17 (0.81; 1.68)	1.19 (0.82; 1.73)	0.347
Adjusted model	1	1.13 (0.78; 1.64)	1.19 (0.81; 1.74)	0.364
Adjusted + BMI	1	1.14 (0.78; 1.65)	1.19 (0.81; 1.74)	0.371
**HDL < 40 mg/dl (*n* = 335)**				
Crude model	1	0.99 (0.73; 1.35)	1.19 (0.88; 1.62)	0.241
Adjusted model	1	1.00 (0.73; 1.36)	1.19 (0.88; 1.62)	0.246
Adjusted + BMI	1	0.99 (0.73; 1.35)	1.19 (0.88; 1.62)	0.247
**LDL ≥ 130 (*n* = 224)**				
Crude model	1	1.17 (0.83; 1.66)	1.03 (0.71; 1.48)	0.878
Adjusted model	1	1.18 (0.83; 1.68)	1.04 (0.72; 1.50)	0.812
Adjusted + BMI	1	1.19 (0.83; 1.69)	1.05 (0.72; 1.51)	0.794
**TG ≥ 150 mg/dl (*n* = 369)**				
Crude model	1	0.91 (0.67; 1.22)	1.16 (0.87; 1.56)	0.295
Adjusted model	1	0.89 (0.66; 1.19)	1.12 (0.83; 1.50)	0.440
Adjusted + BMI	1	0.89 (0.66; 1.20)	1.12 (0.83; 1.50)	0.435
**TC ≥ 200 mg/dl (*n* = 48)**				
Crude model	1	1.02 (0.52; 1.99)	0.68 (0.32; 1.45)	0.337
Adjusted model	1	1.03 (0.53; 2.02)	0.70 (0.33; 1.51)	0.389
Adjusted + BMI	1	1.03 (0.53; 2.02)	0.70 (0.33; 1.51)	0.389
**Abdominal obesity (*n* = 333)**				
Crude model	1	1.30 (0.96; 1.78)	1.30 (0.95; 1.78)	0.106
Adjusted model	1	1.22 (0.89; 1.67)	1.20 (0.87; 1.66)	0.261

MetS, metabolic syndrome; SBP, systolic blood pressure; DBP, diastolic blood pressure; TG, triglyceride; TC, total cholesterol; LDL, low-density lipoprotein; HDL, high-density lipoprotein; FBS, fasting blood sugar.

**Adjusted model:** Variables were controlled for age, gender, socioeconomic status, physical activity, residential region, and screen time. Moreover, MetS components except for abdominal obesity were adjusted for one more confounder (BMI).

-High blood pressure and abdominal obesity cut-offs are based on the 90th percentile or higher based on height, weight, age, and gender. TG, TC, LDL, HDL, and FBS cutoffs were 150, 200, 130, 40, and 100 mg/dL, respectively.

## Discussion

This study investigated the serum level of zinc in children and adolescents and the factors affecting metabolic syndrome. Metabolic syndrome is one of the most significant risk factors associated with cardiovascular disease and type 2 diabetes (3). Studies estimate that more than 100 million children worldwide are obese (16), highlighting the importance of paying attention to metabolic syndrome and its factors in this age group. Moreover, studies also suggest that serum zinc and manganese levels may be essential factors in controlling and synthesizing insulin and controlling fat profile in individuals (17).

This cross-sectional study, based on the population of the Caspian cohort, showed no significant difference in the overall picture between children and adolescents with metabolic syndrome in terms of serum zinc levels. Also, in subgroup analysis, there was no significant correlation between serum zinc levels and age, abdominal obesity, or different fat profiles, and in all evaluations, the numbers did not differ significantly in children with metabolic syndrome or others. However, based on the findings of this study, it seems that healthy girls with metabolic syndrome have lower serum zinc levels than boys. This difference, In addition to biological factors, can also be attributed to the different eating habits of boys and girls. In addition, the studies showed a slight difference between serum zinc levels and blood pressure status in children with metabolic syndrome. Among children with metabolic syndrome, serum zinc levels were significantly lower in children with elevated blood pressure than in others. Compared to this study, few studies have reported a more significant zinc effect than what was seen in this study. For example, in the study of the relationship between serum zinc levels and metabolic syndrome in Korea in 2014, 1,926 people were studied and analyzed. Serum zinc levels were negatively correlated with elevated fasting blood sugar and positively correlated with elevated TG levels in a statistically significant manner. It was also found that in women, serum zinc levels are associated with an increase in the incidence of metabolic syndrome (18). Although, some studies report an association between zinc levels and blood pressure (19), and in theory, zinc could potentially affect blood pressure, due to the effects of zinc on the endothelial cells and vasodilation/constriction through its interactions with various enzymes and proteins (further explained by Tubek) (20); in this study, serum zinc levels were not significantly correlated with blood pressure.

However, it should be noted that the findings on this subject are not entirely consistent. For example, a study by Ghasemi et al. in 2014 in Tehran that was performed on 2401 participants, indicated that there is a significant difference between the genders of participants regarding the relationship between serum zinc levels and metabolic syndrome;(21).

The difference in the populations being studied. (children, adults, specific groups of patients), different adjustments, different settings, confounding variables, and most importantly serum zinc level differences across studies could be the cause of the observed controversial findings. As stated zinc plays a part in many metabolic functions, some of these functions are opposite in nature and outcome (20, 22); Thus many metabolic factors, could potentially impact the results of studies (e.g., mean levels of serum zinc, mean age, sex, ethnicity and other related factors of the population). Hence, more detailed studies are recommended to determine the role of serum zinc levels in the progression of metabolic disease.

It should be noted that in these studies, adults were studied, while in this study, the target population was focused on children and adolescents. Therefore, differences in the final findings are to be expected. In this regard, a study was performed on 60 obese Iranian children by giving zinc and placebo supplements. This intervention showed that zinc supplementation reduces LDL, Total Cholesterol, C-reactive protein (CRP), markers of insulin resistance, fasting blood sugar, insulin, and Homeostatic Model Assessment for Insulin Resistance (HOMA-IR) in children. It should be noted that this study was interventional and prospective, not a cross-sectional one (23).

Our study showed no significant association between serum zinc levels, Mets, and its components. In a similar study conducted on Chinese children and adolescents regarding the association of serum zinc level and metabolic syndrome alongside its components, similarly, no association between serum zinc levels and MetS was found, However serum zinc levels were inversely associated with low-HDL levels and elevated fasting blood glucose with a significant trend (24).

## Recommendation for future research

Further research is needed on the need, deficiency, and biological effects of these supplements to further determine the factors affecting the usefulness of zinc supplementation in children with metabolic syndrome. Furthermore, this study shows that there is no significant difference between children with metabolic syndrome and others in terms of weight; however, the fat profile significantly affected the incidence of metabolic syndrome. Thus, studies need to be conducted, not focusing on obesity but on the global standards of metabolic syndrome in children and adolescents.

## Limitations and strength

To the best of our knowledge, it is the first study that assessed the relationship between serum zinc levels and MetS components, especially with consideration of confounder variables and impressive sample size. However, there are probably some other unknown confounders that are effective in the exact association. Moreover, this study was cross-sectional and due to its natural cause and effect relationship, could not distinguish.

## Conclusion

This cross-sectional study showed no association between serum zinc levels and metabolic syndrome in children. Hence it seems that zinc supplementation in children without zinc deficiency who suffer from Mets or any of its components is redundant. Nonetheless, clinicians must keep racial and age aspects in mind since conflicting findings across different races, sexes, and ages were seen.

Further studies similar studies and cohort studies are needed to reveal the exact relationship between serum zinc levels and metabolic syndrome and warrant our findings.

## Data availability statement

The original contributions presented in this study are included in the article/supplementary material, further inquiries can be directed to the corresponding authors.

## Ethics statement

The studies involving human participants were reviewed and approved by Alborz University of Medical Sciences. Written informed consent to participate in this study was provided by the participants’ legal guardian/next of kin.

## Author contributions

MQ and NM conceived the study. NMK and ED substantially contributed to the conception and design, data analysis and interpretation, drafted the manuscript, and revised it critically. RK, HA, and LS participated in preparing the manuscript. AM-G, ME, and RH participated in the study design and data acquisition. MQ carried out the analysis of the data. All authors read and approved the final version of the manuscript.
